# FOUNTAIN: a modular research platform for integrated real-world evidence generation

**DOI:** 10.1186/s12874-024-02344-w

**Published:** 2024-10-01

**Authors:** Nikolaus G. Oberprieler, Manel Pladevall-Vila, Catherine Johannes, J. Bradley Layton, Asieh Golozar, Martin Lavallee, Fangfang Liu, Maria Kubin, David Vizcaya

**Affiliations:** 1 Bayer AS, Oslo, Norway; 2 RTI Health Solutions, Barcelona, Spain; 3 The Center for Health Policy and Health Services Research, Henry Ford Health System, Detroit, MI USA; 4 RTI Health Solutions, Waltham, MA USA; 5 RTI Health Solutions, Research Triangle Park, NC USA; 6 Odysseus, Inc., New York, NY USA; 7 Bayer AG, Wuppertal, Germany; 8 Bayer Pharmaceuticals, Sant Joan Despi, Spain

**Keywords:** Cohort study, Common data model, FOUNTAIN, Harmonization, Methodology, Real-world data

## Abstract

**Background:**

Real-world evidence (RWE) plays a key role in regulatory and healthcare decision-making, but the potentially fragmentated nature of generated evidence may limit its utility for clinical decision-making. Heterogeneity and a lack of reproducibility in RWE resulting from inconsistent application of methodologies across data sources should be minimized through harmonization.

**Methods:**

This paper’s aim is to describe and reflect upon a multidisciplinary research platform (FOUNTAIN; **F**ineren**O**ne m**U**lti-database **N**e**T**work for evidence gener**A**t**I**o**N**) with coordinated studies using diverse RWE generation approaches and explore the platform’s strengths and limitations. With guidance from an executive advisory committee of multidisciplinary experts and patient representatives, the goal of the FOUNTAIN platform is to harmonize RWE generation across a portfolio of research projects, including research partner collaborations and a common data model (CDM)–based program. FOUNTAIN’s overarching objectives as a research platform are to establish long-term collaborations among pharmacoepidemiology research partners and experts and to integrate diverse approaches for RWE generation, including global protocol execution by research partners in local data sources and common protocol execution in multiple data sources through federated data networks, while ensuring harmonization of medical definitions, methodology, and reproducible artifacts across all studies. Specifically, the aim of the multiple studies run within the frame of FOUNTAIN is to provide insight into the real-world utilization, effectiveness, and safety of finerenone across its life-cycle.

**Results:**

Currently, the FOUNTAIN platform includes 9 research partner collaborations and 8 CDM-mapped data sources from 7 countries (United States, United Kingdom, China, Japan, The Netherlands, Spain, and Denmark). These databases and research partners were selected after a feasibility fit-for-purpose evaluation. Six multicountry, multidatabase, cohort studies are ongoing to describe patient populations, current standard of care, comorbidity profiles, healthcare resource use, and treatment effectiveness and safety in different patient populations with chronic kidney disease and type 2 diabetes. Strengths and potential limitations of FOUNTAIN are described in the context of valid RWE generation.

**Conclusion:**

The establishment of the FOUNTAIN platform has allowed harmonized execution of multiple studies, promoting consistency both within individual studies that employ multiple data sources and across all studies run within the platform’s framework. FOUNTAIN presents a proposal to efficiently improve the consistency and generalizability of RWE on finerenone.

## Background

Real-world evidence (RWE), generated from the analysis of real-world data (RWD), has become a key component of the evaluation of medicinal products. RWE provides insights into the safety and effectiveness of health interventions in the context of routine care, complementing evidence from highly controlled clinical research programs [1, 2]. The utility of RWD for pharmacovigilance and safety-monitoring activities is well established. RWE is becoming increasingly relevant to regulatory agencies, health technology assessment authorities, payers, and medical societies in the evaluation of safe and effective utilization of drugs and devices and the cost-effectiveness of treatment strategies [3–9].

Because of the increased focus on RWE in healthcare decision-making [10], it is essential to ensure that the highest medical, scientific, and ethical standards are upheld during evidence generation. As a consequence, RWE may inform better development, regulatory, and reimbursement strategies. These strategies must integrate scalable, reliable, and agile evidence-generation programs to allow timely communication among stakeholders, ultimately informing evidence-based decisions. Despite a growing focus on RWE within regulatory and healthcare decision processes, its application, impact, and influence have often been hindered by the inconsistency and heterogeneity of the evidence generated [11]. In this regard, several initiatives led by various stakeholders are currently ongoing to inform or provide guidance on the use of RWD or to build infrastructures that enhance the credibility of RWE for decision-making [5–9].

In the past, others have described research platforms designed to support and drive the generation of RWE within pharmacoepidemiologic research [12–16]. These platforms vary in design and execution and are tailored to address a range of research questions and specific objectives. To complement the current landscape of research platforms dedicated to RWE generation, we introduce the FOUNTAIN (FinerenOne mUlti-database NeTwork for evidence generAtIoN) research platform. As part of an ongoing dialogue among the scientific community about best practices for RWE generation, the aims of this paper are to describe and reflect upon FOUNTAIN as a research platform using diverse RWE generation methodologies and to explore the platform’s strengths and limitations.

## Rationale for establishing FOUNTAIN as a research platform

Researchers working with RWD often encounter challenges when conducting and interpreting multidatabase studies. These challenges encompass various factors, including heterogeneity in data collection and management practices, limited data availability and completeness, differences in methodological design, and inconsistencies in terminologies and coding practices across different time periods and geographical regions [10, 11, 17–20]. Moreover, data heterogeneity can arise from intrinsic variations among healthcare systems, guideline recommendations, practice patterns, reimbursement decisions, and cultural contexts. While such heterogeneity is expected and should be accounted for in studies using RWD, it can impact the reliability and validity of the study findings. Therefore, generators of RWE from both public and private sectors must ensure the suitability of data sources for specific research inquiries and apply rigorous methods to address data peculiarities [21]. Additionally, transparent communication of data heterogeneity is crucial during the dissemination of evidence [17]. There are several ongoing initiatives dedicated to establishing best practices for RWD management and evolving methodologies to mitigate data-related hurdles [20].

Similarly, challenges related to the implementation of RWE studies, such as the lack of methodological transparency and reproducibility, have been acknowledged [17–19]. Variation in the application of methodologies across studies can contribute to heterogeneity in RWE results, leading to complex and sometimes divergent interpretations [11, 19]. In addition to managing study-specific sources of bias through careful design and analysis for enhanced internal validity, standardizing and harmonizing methods and analyses can bolster external validity and reproducibility [22]. This, in turn, augments the reliability of RWE for informed decision-making among stakeholders, ultimately enhancing patient care.

Regulatory and health technology authorities have highlighted the need for standardized approaches to promote robustness, consistency, and reliability in the generation of RWE [10, 23, 24]. Of note, the European Medicine Agency’s 2024 guideline on the use of RWD in pharmacoepidemiologic studies was issued to promote harmonization in planning, design, and analysis using fit-for-purpose data in RWE generation [25]. Important research principles that promote the standardization and harmonization of RWE generation methods include the utilization of valid epidemiological and clinical algorithms for identifying study populations, exposures, and outcomes [26]; incorporation of multidisciplinary expertise throughout research activities, from design to interpretation [12]; comprehensive documentation of study methods, encompassing protocols, statistical analysis plans (SAPs), and table shells [27]; and integration of feedback from stakeholders, including patients [28–30], consistently across multiple research programs [12]. An effective approach encompassing these principles could involve the implementation of a research platform centered on a specific research topic.

Accordingly, the overarching rationale for the creation of the FOUNTAIN platform was to generate reliable and consistent RWE to support clinical decision-making and to ensure patient safety for a medication that has recently been launched. The FOUNTAIN platform is a proposed framework to systematically address challenges and limitations of RWE generation by fostering harmonization across individual studies and geographic regions, in turn improving the quality, relevance, and impact of the evidence generated. FOUNTAIN was defined a priori in collaboration with stakeholders, including clinicians, methodology and health economics experts, and patient representatives, to transparently design a flexible evidence generation pipeline that could adapt to the evolution of the dynamic treatment space for conditions, such as chronic kidney disease (CKD) and type 2 diabetes (T2D).

## FOUNTAIN: a modular approach to RWE generation

**Fig. 1:**
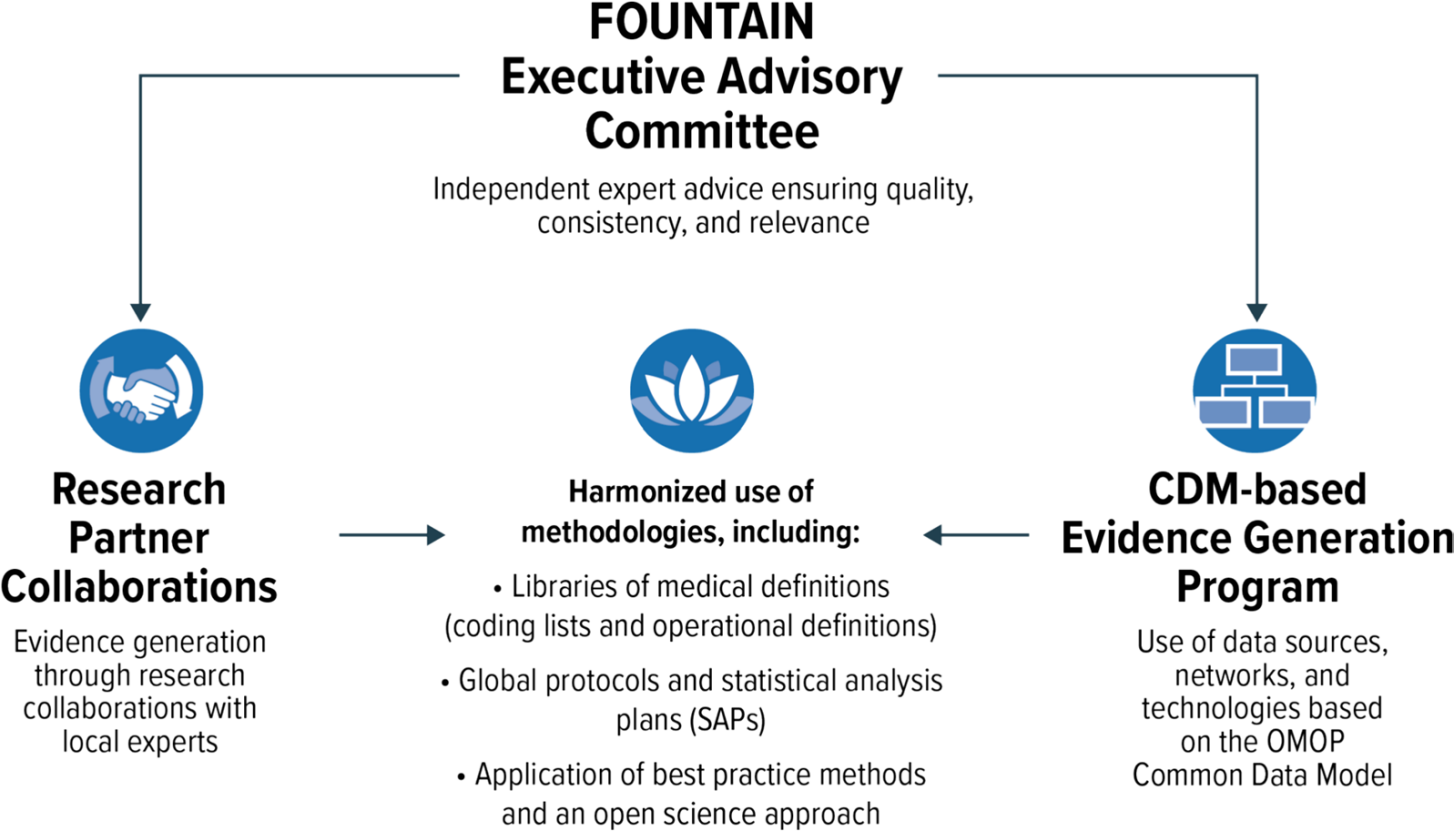
FOUNTAIN Modules and Integration. FOUNTAIN: FinerenOne mUlti-database NeTwork for evidence generAtIoN; OMOP: Observational Medical Outcomes Partnership; CDM: common data model

FOUNTAIN is an integrated approach to RWE generation. The overarching objectives of FOUNTAIN as a research platform are to establish long-term collaborations with pharmacoepidemiology research partners and multidisciplinary experts and to integrate diverse approaches for RWE generation, ensuring harmonization of methodology across all studies. This approach facilitates the integration of extensive clinical, research, and data expertise throughout the implementation of individual studies in a harmonized manner. The primary goal is to enhance the consistency, robustness, reliability, and applicability of the evidence generated. Currently, the aims of the studies run within the frame of FOUNTAIN are to provide comprehensive insight into the real-world utilization, effectiveness, and safety of finerenone, a nonsteroidal mineralocorticoid receptor antagonist newly available as a treatment option for patients with CKD associated with T2D. Finerenone has been recently approved in a number of countries to treat patients with CKD associated with T2D. FOUNTAIN is composed of three main modules: an executive advisory committee (EAC) comprising multidisciplinary experts and patients; a series of partnerships with international research and data institutions to ensure knowledge of local health systems and practices; and a separate, federated evidence-generation approach based on the use of a common data model (CDM) with rapid scalability (Fig. 1).

FOUNTAIN is intended to facilitate cost-effective, timely, and targeted evidence generation by uniting research and data partners, investigators, international multidisciplinary experts, patients, and industry representatives in a coordinated effort. From a structural perspective, the research platform enables a harmonized application of best-practice methodologies by using libraries of medical definitions (code lists and operational definitions) and global protocols and SAPs. FOUNTAIN adheres to an open science approach, with a commitment to fully transparent methodologies, consistent use of definitions, public registration of protocols, and comprehensive and timely publication of results. Furthermore, this platform combines two complementary approaches to multicountry, multidatabase RWE generation. In one approach, research partners and investigators lead the execution of a global protocol and SAP in their native data source locally. In conjunction with all research collaborators, the protocol and SAP are harmonized to encompass the unique attributes of each data source, thereby ensuring replicable and resilient execution of all analyses within each data source. In the other approach, a centralized execution of a common protocol and SAP in multiple data sources is implemented through federated data networks mapped to the same CDM. CDM-based research implements analyses via a standardized analytical approach executed across multiple data sources that are mapped to a CDM [31]. This approach usually includes the use and repurposing of existing analytical tools to address the research objectives of interest.

Currently, FOUNTAIN supports multiple studies within two research lines: (1) characterization, drug utilization, and treatment patterns in patients with CKD and T2D; and (2) finerenone safety and effectiveness in clinical practice [32]. In the sections that follow, we describe the FOUNTAIN research modules and the research programs currently ongoing within the platform.

### Executive advisory committee

FOUNTAIN benefits from an EAC that provides guidance on the design and execution of the research programs, especially for those studies relevant for health authorities. FOUNTAIN’s EAC is a multidisciplinary group of international experts in the fields of clinical research, health economics, and epidemiology, as well as patient representatives. The EAC reviews research questions for the different research programs under FOUNTAIN to ensure that they are precisely defined while addressing the requirements of a broad spectrum of stakeholders and advises on the use of appropriate and fit-for-purpose methods to address the specific objectives of each program. The EAC also plays a critical role in assisting with the appropriate interpretation of study findings and in preparing the findings for application by relevant stakeholders, ensuring that research outcomes are effectively communicated and utilized within the broader healthcare community.

### Research collaborations for evidence generation

Establishing a multidisciplinary research platform necessitates identifying the platform’s specific requirements to address specific research questions. In the context of the FOUNTAIN research platform, the specific research projects aim to provide comprehensive insight into the real-world utilization, effectiveness, and safety of finerenone.

Designing a comprehensive research program using multiple research and data partnerships utilizing a common protocol requires careful planning before the research is initiated [33, 34]. Depending on the regulatory and legal requirements and the research objectives, initial landscaping may include practical considerations, such as identifying the countries in which the product will be marketed, anticipated timing of product launch and reimbursement, and anticipated uptake of the product in specific countries [17, 35, 36]. In addition, this planning phase requires a thorough evaluation of existing clinical practice for treatment of the condition for which the new product is indicated, thereby facilitating the identification of possible comparator groups for safety and effectiveness research [33, 34, 37–39].

After this initial step, a feasibility evaluation conducted in the target countries to evaluate appropriate data sources and identify data holders with whom research partnerships can be established is crucial. Selection of fit-for-purpose data sources (in terms of quality of the data, relevance of the data source for the research question, and data access) requires knowledge of the study objectives and anticipated study size to determine whether multiple data sources are needed and what types of data are needed to answer the research questions. A thorough feasibility evaluation of data sources includes review of publicly available information and contact with data holders to answer more detailed questions about data availability [21]. In the context of FOUNTAIN, a thorough database feasibility evaluation was conducted in 14 preselected databases in the European Union, North America, and Asia, focusing on criteria, such as general data quality, data completeness, and availability of outpatient laboratory measurements.

In a research collaboration using multiple databases with local research partners, analyses are implemented separately by each research partner in a harmonized way with a common study protocol and SAP, adapted to each specific database, and developed with input from research partners and study investigators. With this approach, heterogeneity among different healthcare systems, data sources, and data types is permitted in the interest of producing high-quality evidence that is tailored to and meaningful for clinical practice in individual settings. For example, differences in healthcare systems and database types between US-based and European data sources often demand adapted operationalizations of the same scientific concept. To ensure a good harmonization across data sources, study documentation must be clear, transparent, and unambiguous [27].

This type of collaboration ensures flexibility and transparency [40, 41] and adapts the research plan to the realities and intricacies of each data source, such as disease coding systems and coding practices, availability of specific data elements (e.g., laboratory results; linkage to other data, such as disease-specific registries and hospital discharge diagnoses), and availability of drug dispensing versus prescription information. Collaborating with local research institutions also allows consideration and integration of local expertise in clinical practices and database specificities in the research [42]. This approach requires substantial planning and implementation time and careful oversight, and it is usually more costly compared with single-study projects and other approaches due to the need for multiple investigators and site-specific analysts.

### Evidence generation using data sources mapped to a CDM

In coordination with the FOUNTAIN Executive Advisory Committee and pharmacoepidemiologic research partners, CDM-based evidence generation stands as the third module of the FOUNTAIN research platform. The ability to leverage data sources mapped to a CDM facilitates rapid evidence generation within the FOUNTAIN framework, bolstering both agility and scalability in evidence generation.

Research logic in the context of a CDM-federated data network is slightly different from the research collaboration approach described above. Although CDM-based research utilizes the same concepts of a global protocol and SAP and multidisciplinary teams of researchers and experts from different healthcare databases, it extends the harmonization to a standardized data model that enables efficient integration of diverse healthcare data sources. This facilitates large-scale analyses across multiple institutions and fosters interoperability, leading to improved reproducibility and comparability of results. The standardized format enhances the feasibility of conducting comprehensive characterization, comparative effectiveness, and post-market safety studies using multiple databases.

Mapping a database to the CDM is achieved through the standardization of the data source structure and vocabularies in a process known as extract, transform, and load (ETL). This process ensures that the resulting data structure is compatible with the target CDM—for example, Observation Medical Outcomes Partnership (OMOP). Subsequently, utilization of a CDM allows researchers to implement a ubiquitous method across a network of data sources that was mapped to that CDM. The potential role and importance of CDM-based observational research has been widely discussed [36], and the impact and speed of generating RWE using CDM-based approaches has been demonstrated during the coronavirus disease 2019 (COVID-19) pandemic [43].

Currently, various CDMs are available in observational health research. The open-source, nonproprietary OMOP CDM is the most widely used globally and across research networks and is being adopted by regulatory agencies, including the US Food and Drug Administration and the European Medicines Agency [6, 8, 44]. The OMOP CDM was originally developed through a public–private partnership involving multiple collaborators from academia, government, and industry, including the US Food and Drug Administration, pharmaceutical companies, and healthcare providers [45].

The introduction of a new drug often triggers new research questions throughout its life cycle. Supporting regulatory submissions for new drugs or new indications by complementing clinical trial evidence with RWE or supporting clinical development plans with contemporary evidence on conditions of interest are examples related to research and development at the industry level [46]. Similarly, use of the product in the real world may raise questions from authorities that often require a rapid response from the market authorization holder. These aspects would benefit from proactive and even anticipatory evidence-generation capabilities from the research community. For these reasons, we designed a program to leverage data sources mapped to a CDM to facilitate rapid and nimble evidence generation within the FOUNTAIN framework. This program relies on the OMOP CDM, best practices, and open-source analytical tools from the Observational Health Data Sciences and Informatics (OHDSI) scientific community [47, 48] and the medical expertise and harmonization provided by FOUNTAIN. It creates an ecosystem in which many research questions can be addressed rapidly through analytical interfaces and tools, such as ATLAS [49] and the HADES suite of tools [50, 51], and can efficiently be scaled up to additional databases or data networks by sharing ready-to-use study packages.

Currently, the CDM-based evidence generation program includes studies intended to provide additional granularity and scope to enhance and contextualize evidence generated in the research partner collaboration programs. Specific aims include exploring the heterogeneity of subgroups of different user cohorts, supplementing evidence regarding healthcare resource utilization, expanding the scope of drug utilization studies, and providing insights on the effect of the timing of cohort entry along the timeline of disease progression. To ensure sustainability, the CDM-based evidence generation approach will also support clinical development programs for related indications and other therapeutic areas and, potentially, generate evidence about the safety and effectiveness of treatment options. In addition, we will generate an open-access library of cohort definitions and phenotypes using standardized vocabulary from OMOP CDM that are harmonized under FOUNTAIN’s umbrella. These definitions will be available for the scientific community and ready to be repurposed for generating or refining evidence in a variety of data sources and healthcare systems.

## Strengths and limitations of the FOUNTAIN platform

**Fig. 2:**
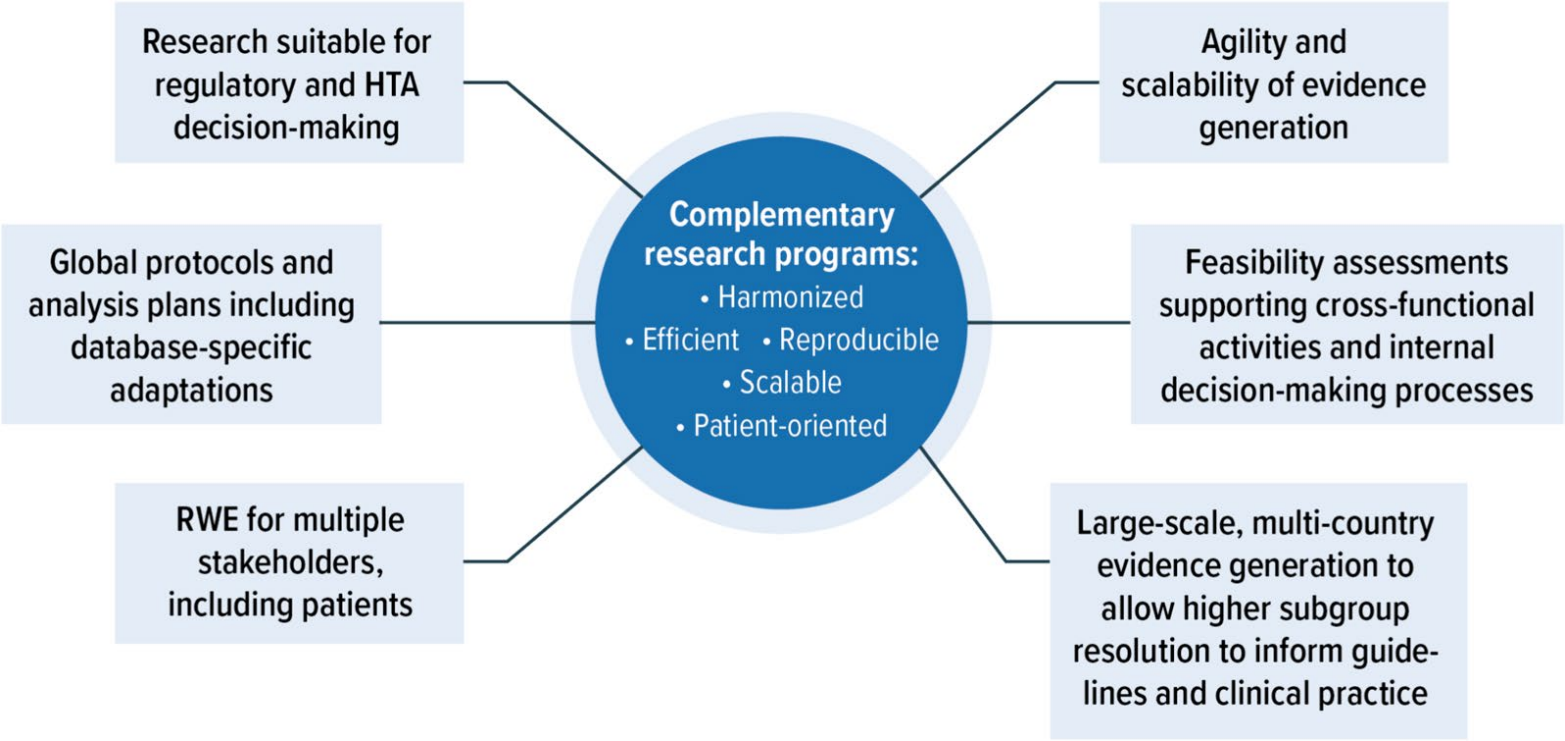
FOUNTAIN—an Integrated, Modular Research Platform. RWE: real-world evidence; HTA: health technology assessment

Bringing together patients, methodological and process experts, research and data partners, and academic and commercial collaborators in one harmonized platform provides the foundation for the generation and delivery of robust and timely RWE (Fig. 2). Proactive planning to ensure alignment on choice of data sources, protocols, definitions, methodologies, and research approaches across all partners enables the initiation and execution of multiple research initiatives in a robust and agile manner. Furthermore, due to the systematic harmonization across individual studies and evidence generation approaches, the results generated through the FOUNTAIN platform can be interpreted and contextualized across studies and geographies.

The methodologic details of the individual studies run within the FOUNTAIN platform are described elsewhere [52–54]. Briefly, the goal of the FINEGUST study was to map and describe treatment patterns and drug utilization in patients with CKD and T2D (NCT05526157) [52, 55, 56]. Following the principles described of the FOUNTAIN platform, the FINEGUST harmonized protocol for a multicountry research partner collaboration was leveraged to expand the scope (additional medication cohorts, databases, comorbidity subgroups) using the CDM-based evidence generation approach [57]. This allowed for an efficient, harmonized, and consistent description of treatment patterns across databases and geographic regions. A similar approach was followed for the safety and effectiveness program within FOUNTAIN (NCT06278207) [53, 54, 58].

Many elements of a multidisciplinary platform, such as FOUNTAIN, can be established through detailed planning, alignment, and collaboration, but some limitations must be considered before initiating such a research approach. For example, harmonization of global research protocols and standardized study execution in different countries and healthcare systems can be limited by differences in local legislation and regulations. These differences have the potential to affect a range of research activities, such as protocol approvals, ethics review committees and requirements, data access, quality control by international collaborators, and publication of results. Furthermore, differences in data source–specific processes and operations can hinder optimal standardization across different data partners. For instance, lag times for data availability can vary due to diverse data-collection routines in respective data sources; this becomes especially relevant when the medication of interest is a newly available treatment option. Differences in guideline recommendations, reimbursement decisions, and clinical implementation of a new treatment option in different healthcare systems or countries can potentially further compound issues of data availability.

Using a standardized analytical package to execute a single protocol across a federated network of data sources for RWE generation has a number of advantages, as discussed. However, application of this approach requires careful consideration. As an example, the differential validity of a programmed case ascertainment algorithm across different data sources and healthcare systems may impact the validity of the overall analysis. It is therefore critical to stringently review and revise algorithms to ensure their validity across all data sources. Despite the effort in creating efficiencies in observational research conduct, there are limitations in CDM approaches. For example, a CDM approach involving multiple databases requires a strong coordination to overcome differences in technical environments, ethical requirements, and timings to ensure levering its full potential when it comes to federated data initiatives. Likewise, the systematic analytic execution of a single script across multiple databases makes it difficult to adapt the analysis to data-specific challenges. To overcome this, multiple diagnostics are commonly implemented to select the appropriate research methods and databases as a best practice [50]. Finally, the use of a CDM approach often reduces the methodological options applicable. However, organizations, such as OHDSI, have developed large suites of open-source statistical packages including a broad number of cutting edge statistical and epidemiological methods [59]. When not done carefully, the ETL process may incur a nonnegligible loss of information through, for example, poor mapping specifications. However, there is evidence that any potential loss of information is minimal and does not affect the accuracy and consistency of the evidence generated from an epidemiological and statistical standpoint [60–63]; use of a CDM ETL process also ensures data cleaning and debugging of data errors [64].

Establishing and maintaining a research platform like FOUNTAIN undoubtedly requires more upfront investment in planning and coordination than a single-study project. However, harmonization of RWE generation across different research programs will strengthen the external validity of the generated evidence, enhancing its value and relevance to support clinical decision-making. Furthermore, the key features of coordinated platforms like FOUNTAIN position research teams to address new and evolving research questions in an anticipatory and timely manner, which can be transferred to support evidence generation across different indications and therapeutic areas.

To exemplify the practical implementation of the approaches described above, we present two examples of study programs within the FOUNTAIN platform. Understanding the dynamic treatment landscape for the target indication for a new drug, such as finerenone, is foundational in evaluating its effectiveness and safety. To provide this background knowledge, a specific research program focuses on describing utilization patterns of medications with proven or potential benefits for preventing deterioration of renal function among patients with CKD associated with T2D, before and after the introduction of finerenone, in clinical practice in countries in Europe (Denmark, The Netherlands, and Spain), the United States (US), China, and Japan [52]. Additionally, as part of the wider treatment patterns program, standalone studies utilizing the CDM approach, designed on the basis of harmonized methods with FOUNTAIN criteria (e.g., cohort definitions, fit-for-purpose database), are ongoing to explore more specific research questions to address different aspects on treatment patterns as part of standard of care in patients with CKD associated with T2D.

Besides understanding baseline characteristics and treatment patterns, it is important to understand patients’ clinical course and how it is affected by the addition of a new treatment option. Safety and effectiveness studies aim to provide such evidence, and a second comprehensive research program has been designed to evaluate the impact of finerenone on health outcomes in routine clinical practice [53]. Estimating the safety and effectiveness of finerenone requires application of complex analytical and study design methods, and their assumptions and potential biases need to be considered when interpreting results. Therefore, this program is being implemented in a staggered manner, with an initial goal of understanding clinical outcomes in patient cohorts before the new drug of interest becomes available. Complementary studies are aiming to provide similar evidence in patients using finerenone after its launch and will explore potential comparators for future effectiveness and safety analyses [53]. The ultimate goal of this staggered approach is to prepare for a series of inferential, comparative studies evaluating the real-world safety and effectiveness of finerenone in the US and internationally in a scientifically robust manner, informed by the findings of the earlier studies. To provide further insights on the real-world effectiveness and safety of finerenone in clinical practice, individual studies utilizing the CDM approach are closely integrated in this program to address specific evidence gaps.

## Conclusion

As the demand for robust RWE increases across a multitude of stakeholders, the scientific community needs to find efficient ways of improving the consistency and generalizability of the evidence being generated. In contrast to more traditional clinical research settings, standards for RWE generation are more multifaceted, thus requiring a heterogeneous, portfolio-like approach to generate comprehensive and actionable evidence. To improve the impact of RWE on clinical practice, decision processes and, ultimately, patient care approaches to RWE generation have to continually evolve. In the context of establishing the FOUNTAIN modular research platform, its strengths, including diverse analytical approaches and mitigated inconsistencies in RWE generation, have been outlined here, alongside potential limitations. FOUNTAIN presents a proposal to efficiently improve the consistency and generalizability of RWE on finerenone.

## Data Availability

Not applicable.

## Abbreviations

CDM: Common data model
CKD: Chronic kidney disease
COVID-19: Coronavirus disease 2019
EAC: Executive advisory committee
ETL: Extract, transform, and load
FOUNTAIN: FinerenOne mUlti-database NeTwork for evidence generation
OHDSI: Observational Health Data Sciences and Informatics
OMOP: Observation Medical Outcomes Partnership
RWD: Real-world data
RWE: Real-world evidence
SAP: Statistical analysis plan
SPRING: SupPoRt system for characterization and INsights Generation in Kidney disease
T2D: Type 2 diabetes
US: United States
